# High prevalence of extrapyramidal signs and symptoms in a group of Italian dental technicians

**DOI:** 10.1186/1471-2377-7-24

**Published:** 2007-08-08

**Authors:** Edito Fabrizio, Nicola Vanacore, Marcella Valente, Alfonso Rubino, Giuseppe Meco

**Affiliations:** 1 Department of Neurological Sciences, "La Sapienza" University, Viale Università 3000185 Rome, Italy; 2 National Centre of Epidemiology, National Institute of Health, Via Giano della Bella 34, 00161 Rome, Italy

## Abstract

**Background:**

Occupational and chronic exposure to solvents and metals is considered a possible risk factor for Parkinson's disease and essential tremor. While manufacturing dental prostheses, dental technicians are exposed to numerous chemicals that contain toxins known to affect the central nervous system, such as solvents (which contain n-hexane in particular) and metals (which contain mercury, iron, chromium, cobalt and nickel).

**Methods:**

We performed an epidemiological and clinical study on all 27 dental technicians working in a school for dental technicians. We asked all the technicians to fill in a self-administered questionnaire on extrapyramidal symptoms, and the General Health Questionnaire (GHQ), a self-administered screening instrument, to detect any psychiatric disorders. Moreover, we invited all 27 dental technicians to undergo a neurological examination and provide a detailed occupational history in our clinic.

**Results:**

Of the 14 subjects who underwent the neurological examination, four had postural tremor and one had a diagnosis of Parkinson's disease.

**Conclusion:**

We found a high prevalence of extrapyramidal signs and symptoms in this group of male dental technicians working in a state technical high school in Rome. We believe that this finding may be due to the presence of toxins in the dental technician's work.

## Background

Occupational and chronic exposure to solvents and metals is considered a possible risk factor for Parkinson's disease (PD).

Numerous case-control studies point to a role of these environmental toxins in the etiopathogenesis of PD.

One study performed on 144 PD cases and 464 controls revealed a higher incidence of PD among subjects with more than 20 years' exposure to lead-iron (OR = 2.83; 95CI% 1.07–7.50) and iron-copper (OR = 3.69; 95 CI% 1.40–9.71) combinations [1].

Another study, conducted on 341 cases and 357 controls, revealed that the incidence of PD was associated with occupational exposure to solvents (OR = 1.89; 95 CI% 1.2–2.7) [2].

Yet another analytical epidemiological study indicated a possible association between exposure to mercury and PD [3]. Moreover, some case reports point to a link between parkinsonism and exposure to n-hexane and mercury [4,5]. An epidemiological review of this topic has been published [6]. Other case-control studies report contrasting results regarding a possible link between occupational exposure to metals and solvents and essential tremor (ET) [7,8]. Dental technicians are exposed to dust and vapour during the manufacture of dental prostheses from the negative impression of the patients' teeth provided by the dentist. Dental production entails exposure to various chemical hazards, including solvents, mineral acids, gases and vapours released during polymerisation, metal casting and porcelain baking, as well as to dust from plaster, metal alloys, ceramics and acrylic resins [9]. Moreover, during the processing of resins, particles of dust of methacrylate compounds are worked with oxidative substances that release free radicals. The dust also contains inorganic pigments such as mercury sulphide. Dental technicians are consequently exposed during the manufacture of dental prostheses to numerous chemicals that contain toxins known to affect the central nervous system, such as solvents (which contain n-hexane in particular), metals (which contain mercury, iron, chromium, cobalt and nickel) and bisphenol-A [9]. In this study, we found a high prevalence of extrapyramidal symptoms in a group of male dental technicians working in a state technical high school in Rome.

## Methods and population

A 47-year-old, right-handed male was referred to our specialized centre for the diagnosis and treatment of PD and other extrapyramidal diseases. The neurological symptoms in this patient, consisting of heaviness in the right arm, had first appeared at the age of 44 years. At the clinical examination, the patient scored 13 in the UPDRS motor scale, (speech 1, facial expression 2, right-arm postural tremor 1, neck rigidity 1, right-arm rigidity 2, right-leg rigidity 1, right-hand finger taps 1, right-hand movements 1, right-hand rapid alternating movements 2, gait 1). The patient's anamnesis did not reveal a family history of either parkinsonism or ET.

Magnetic resonance images of the encephalon, the electromyography, sensorimotor nerve conduction, motor evoked potential, serum copper and serum ceruloplasmin were all normal. An acute L-dopa test, in which the patient took one tablet of levodopa and carbidopa at dosages of 250 and 25 mg, respectively, improved the UPDRS motor score after two hours by 46%, reducing it from 13 to 7. The patient is now 50 years old and, 7 years after the onset of symptoms, and 3 years after first coming to our observation, during which the disease has remained clinically stable, the parkinsonism continues to be strictly lateralized on the right side (UPDRS motor score of 12). He has, for the last 16 months, been taking antiparkinsonian drugs; he is currently on pramipexole and selegiline at dosages of 2.1 mg/day and 5 mg/day, respectively.

Although he has had a right hemiparkinsonism for 7 years, our patient's other clinical features meet the UK Brain Bank clinical diagnosis criteria for probable PD.

He is a dental technician who had been teaching in a state school for dental technicians in Rome.

He had, for the previous 30 years, always worked in an environment in which numerous toxic substances, such as mercury sulphate, metals and solvents, were used. The patient had never used any protective clothing, nor had he ever undergone any biochemical tests to assess his level of exposure to solvents and metals.

Moreover, the patient referred that many of his colleagues had similar symptoms.

We performed an epidemiological and clinical study on all 27 dental technicians (including the above patient) working in the same school for dental technicians.

We asked all the technicians to fill in a self-administered questionnaire on extrapyramidal symptoms, which is used as a screening instrument to detect parkinsonism cases in the general population [10]. This questionnaire contained nine questions on symptoms (presence or absence of a symptom) and two questions regarding the diagnosis and treatment of PD. Using a cut-off of four positive answers, this questionnaire achieves a sensitivity of 90% and a specificity of 94% [10].

We also used the General Health Questionnaire (GHQ), a self-administered screening instrument, to detect any psychiatric disorders [11]. The GHQ is designed to cover four identifiable elements of distress: depression, anxiety, social impairment and hypochondriasis (indicated above all by organic symptoms). We used a GHQ-28 item version. Items were scored using conventional 0,1,2,3 Likert scores for the response categories. When a two-point score (present or absent) was used for the GHQ, a cut-off score of 6 or above was the optimal threshold for sensitivity (79.2%) and specificity (79.6%)[11].

Lastly, all 27 dental technicians were invited to undergo a neurological examination and provide a detailed occupational history in our clinic.

This study was approved by the local ethical committee, and written consent was obtained from all subjects.

### Statistical analysis

A student's t-test was used for the comparisons. The frequency analysis was performed by means of the chi-square test. A p value of 0.05 was considered statistically significant. All the statistical analyses were carried out by means of SPSS software (Version 13.0).

## Results

The 27 male dental technicians had a mean age of 49.1 ± 5.5 years (range 41–64 yrs) and a mean duration of exposure to toxins in dental work of 24.7 ± 3.1 years (range 17–31 yrs).

Twelve subjects responded positively to at least four questions in the self-administered questionnaire on extrapyramidal symptoms. Fifteen subjects has a score of 6 or above in the GHQ. Fourteen subjects accepted the invitation to undergo a clinical evaluation (neurological examination and administration of UPDRS) and provide an accurate occupational anamnesis in our clinic.

There were no significant differences in age, duration of exposure to toxins, positive screening for extrapyramidal symptoms or the GHQ total, subtotal and cut-off scores between the 14 subjects that came to the clinic and the 13 that did not (Table 1).

**Table 1:** Comparisons between the two groups of dental technicians included in the study

**Variables**	**Subjects who underwent neurological examination (n = 14)**	**Subjects who did not undergo neurological examination (n = 13)**	**p value**
Mean age (years)	50 ± 5.9	48.1 ± 5	n.s.
Mean exposure to dental work (years)	25.6 ± 3	24.0 ± 3.1	n.s.
Subjects with at least four positive answers in questionnaire for parkinsonism (y/n)	7/7	5/8	n.s.
GHQ* mean total score	28.7 ± 14.9	26.0 ± 12.2	n.s.
Somatic symptoms mean score (GHQ)	8.0 ± 4.4	7.0 ± 4.5	n.s.
Anxiety mean score (GHQ)	8.4 ± 5.2	8.0 ± 5.1	n.s.
Social dysfunction mean score (GHQ)	9.8 ± 3.7	8.2 ± 1.4	n.s.
Depression mean score (GHQ)	2.5 ± 4.2	2.8 ± 2.6	n.s.
Subjects with a total score of 6 or above in the GHQ (y/n)	8/6	7/6	n.s.

* The General Health Questionnaire

The proportion of subjects examined by us at the clinic was greater among subjects who reported shaking in the arms or legs in the questionnaire than those who did not (8/6 vs 2/11; p = 0.02).

Of the 14 subjects who underwent the neurological examination, four had postural tremor and one PD (i.e. the aforementioned patient described in detail). The age, clinical and occupational variables of these five dental technicians are shown in Table 2.

**Table 2:** Clinical and occupational characteristic of five dental technicians with extrapyramidal symptoms

**Patient**	**Age**	**Age at onset**	**Diagnosis**	**Family history of PD and/or essential tremor**	**UPDRS motor score**	**Exposure to dental work (years)**
1	50	44	Parkinson's disease	No	12	30
2	64	62	Bilateral postural tremor	No	2	25
3	42	38	Bilateral postural tremor	Yes	2	22
4	56	46	Bilateral postural tremor	No	3	31
5	52	46	Unilateral postural tremor	No	1	23

From a clinical point of view, three subjects had bilateral postural tremor, while one had monolateral postural tremor [12]. One subject had postural and kinetic tremor. The tremor in all four subjects was mild, low amplitude, barely perceivable or intermittent. Only one subject had a positive family history of postural tremor.

Two subjects (49 and 46 years old) with a positive screening for parkinsonism and shaking in the arms or legs, according to the self-administered questionnaire, were both found to be negative at the neurological examination, even though the 49-year-old subject as well as his family reported postural and kinetic tremor.

Among the subjects that underwent the neurological examination, there were no statistically significant differences in age, duration of exposure to toxins, GHQ total, subtotal and cut-off scores between subjects with neurological signs and those without (data not shown); the only statistically significant difference between these two groups was in the number of subjects with at least four positive answers in the questionnaire on extrapyramidal symptoms (5/1 vs 2/6; p= 0.03).

## Discussion

We found a high prevalence of extrapyramidal signs and symptoms in a group of dental technicians working in a state technical high school in Rome (one subject with PD, three with bilateral postural tremor and one with monolateral postural tremor).

Although the mean age of the dental technicians included in this study was not very high (49.1 ± 5.5 yrs), the frequency of bilateral postural tremor (3/27 = 11.1%) and PD (1/27 = 3.7%) in this group were markedly higher than those reported in epidemiological studies on these pathologies (0.4–3.9% for ET and 0.17–0.25% for PD)[12].

Disease progression in our patient with sporadic PD was atypical since his hemiparkinsonism had been present for seven years and had been stable for at least three years. This clinical picture has frequently been associated with parkinsonism of probable toxic origin, though a genetic origin cannot be ruled out.

The proportion of ET cases with a family history of ET ranges from as low as 17% to as high as 100% [12]. In this study, only one subject had a familiar history of ET (1/27 = 3.7%).

All five dental technicians displaying extrapyramidal signs at the neurological examination had been exposed for many years to toxins known to affect the extrapyramidal system (solvents and metals, particularly mercury and n-hexane, and chemicals such as para-nonylphenol and bisphenol-A) that significantly stimulate hydroxyl radical formation in the striatum [13]. Moreover, the technicians worked in a particularly difficult environment in which there was inadequate ventilation, insufficient light, a large number of people (up to 25) working in an area of 30–40 metres squares, and excessive noise due to the use of drills, vibrators, mixers and a casting machine.

We also examined a 44-year-old male dental technician who had worked in this school for 16 years. This subject had bilateral postural tremor that first appeared approximately 10 years ago.

We cannot exclude the presence of other cases with such pathologies among the dental technicians who tested positive in the screening phase for parkinsonism but refused to undergo the clinical examination.

There are several limitations in our study that need to be borne in mind: a) the absence of a control group prevents us from comparing the extrapyramidal signs and symptoms in our group of dental technicians with those in the general population; b) we based our data on the presence of known neurotoxins in the dental technicians' school from information on the labels of the toxicological substances used, without assessing the exposure level or biological markers of neurotoxins present in the workplace itself; c) we did not formally screen the subjects for ET, though the self-reported questionnaire on extrapyramidal symptoms does include a specific item on tremor.

## Conclusion

On the basis of the epidemiological, occupational and clinical data described above, we believe that the high prevalence of extrapyramidal signs and symptoms found in these 27 dental technicians working in the same school was not a chance occurrence, but was probably related to the presence of known neurotoxins in the dental workers' profession.

In Italy, there are estimated to be approximately 1000 dental technicians working in 30 state technical high schools and 15 000 dental technicians working in 5000 dental laboratories.

An epidemiological study designed to assess the possible risk of parkinsonism, PD and ET in a large cohort of dental technicians is warranted.

## Competing interests

The author(s) declare that they have no competing interests.

## Authors' contributions

EF conducted the clinical and epidemiological study and conceived the study;

NV participated in the design of the study and performed the statistical analysis;

MV conducted the clinical study;

AR conducted the clinical study;

GM conceived the study, participated in its design and coordination and helped to draft the manuscript.

All authors read and approved the final manuscript.

## Pre-publication history

The pre-publication history for this paper can be accessed here:


